# Hierarchical Amplitude-Aware Permutation Entropy-Based Fault Feature Extraction Method for Rolling Bearings

**DOI:** 10.3390/e24030310

**Published:** 2022-02-22

**Authors:** Zhe Li, Yahui Cui, Longlong Li, Runlin Chen, Liang Dong, Juan Du

**Affiliations:** 1 School of Mechanical and Precision Instrument Engineering, Xi’an University of Technology, Xi’an 710048, China; 1200211012@stu.xaut.edu.cn (Z.L.); cyhxut@xaut.edu.cn (Y.C.); chenrunlin@xaut.edu.cn (R.C.); 1200213020@stu.xaut.edu.cn (L.D.); 2 Department of Basic, Air Force Engineering University, Xi’an 710051, China; juandu@stu.xidian.edu.cn

**Keywords:** hierarchical amplitude-aware permutation entropy, rolling bearing, performance trend state assessment, fault feature extraction

## Abstract

In order to detect the incipient fault of rolling bearings and to effectively identify fault characteristics, based on amplitude-aware permutation entropy (*AAPE*), an enhanced method named hierarchical amplitude-aware permutation entropy (*HAAPE*) is proposed in this paper to solve complex time series in a new dynamic change analysis. Firstly, hierarchical analysis and *AAPE* are combined to excavate multilevel fault information, both low-frequency and high-frequency components of the abnormal bearing vibration signal. Secondly, from the experimental analysis, it is found that *HAAPE* is sensitive to the early failure of rolling bearings, which makes it suitable to evaluate the performance degradation of a bearing in its run-to-failure life cycle. Finally, a fault feature selection strategy based on *HAAPE* is put forward to select the bearing fault characteristics after the application of the least common multiple in singular value decomposition (LCM-SVD) method to the fault vibration signal. Moreover, several other entropy-based methods are also introduced for a comparative analysis of the experimental data, and the results demonstrate that *HAAPE* can extract fault features more effectively and with a higher accuracy.

## 1. Introduction

The condition monitoring of rotating machinery is a fundamental task in prognostics and health management systems [1]. As an essential part of rotating machinery, rolling bearings are critical and more easily damaged components compared to others [2]. If a rolling bearing has a local defect on the matching surface, a series of shocks are generated due to periodic collisions when the rolling element rolls over the defect area. These abnormal shocks may result in an unexpected failure of large-scale equipment if without timely inspection and maintenance [3,4]. To ensure the reliable operation of rolling bearings, a degradation evaluation of its running state and a fault diagnosis, two indispensable aspects of modern advanced rotating equipment, can be employed. Additionally, with these two techniques, considerable maintenance costs can be saved. Therefore, the performance evaluation and fault diagnosis of rolling bearings have become a major topic of research.

Nowadays, in the condition monitoring of rotating machinery, vibration-signal-based processing techniques are one of the most used methods for fault diagnosis, which can be classified into three categories: time-domain analysis (e.g., root mean square (RMS) and kurtosis), frequency-domain analysis (e.g., fast Fourier transform and envelope spectrum demodulation), and time–frequency analysis [5]. In addition, time–frequency analysis has become a popular method for rolling bearing fault feature extraction, and wavelet transform (WT) [6], empirical mode decomposition (EMD) [7], ensemble empirical mode decomposition (EEMD) [8], and variational mode decomposition (VMD) [9] are several typical time–frequency analysis algorithms. However, the parameter selection of the above methods has some influence on their performance [10,11,12]. By contrast, as a matrix decomposition method, singular value decomposition (SVD) is a nonparametric algorithm that has been widely used in the noise reduction field [13,14]. The denoising method based on SVD eliminates the noise and defect unrelated components by setting the singular values (SVs) corresponding to the noise-related components to 0; thus, the informative components can be reconstructed from the persevered SCs [15]. The critical issue of reconstruction is to determine the dimension of the Hankel matrix. Generally, the dimension of the Hankel matrix can be reconstructed using the maximum dimension method, which depends on the length of the signal but ignores the signal frequency characteristics. The least common multiple in SVD (LCM-SVD) method has been introduced to address this problem, and it determines the reconstructed matrix’s dimension based on frequency factor analysis [16].

Regarding the nonlinear dynamic analysis method of rolling bearing signals, entropy is developed into an effective approach to measure the dynamic change in complex time series in fault diagnosis [17]. Sample entropy (SE), fuzzy entropy (FE), and permutation entropy (PE) [18] algorithms are several entropy-based indexes that are extensively employed. Compared with the SE and FE approaches, PE has many advantages, such as clean theory, fast calculation efficiency, and robust anti-noise performance [19]. PE is always used to estimate the different patterns of orderings that are scattered in a time series and plays a significant role in feature extraction [20]. However, the direct application of PE in feature extraction does not ensure the data’s denoising effect; hence, it is often used after a noise removal method [21]. Apart from this, PE is disabled to assess the amplitude information of the signal. To settle this issue, Azami et al., proposed an entropy algorithm named amplitude-aware permutation entropy (*AAPE*) based on PE, which improves its sensitivity to the amplitude and frequency of the time series by using the counting rule for each sorting mode [22,23]. However, single-scale entropy, such as SE, FE, PE, and *AAPE*, may lose key information during time series analysis. To solve this problem, multi-scale entropy (MSE) was first proposed by Costa M. et al., to measure the complexity of a time series [24]. Subsequently, multi-scale fuzzy entropy (MFE) [25], multi-scale permutation entropy (MPE) [26], and multi-scale amplitude-aware permutation entropy (MAAPE [27]) and its variants (IMAAPE [28]) were proposed sequentially. However, multi-scale entropy analysis ignores the effect of high-frequency components. To obtain more helpful information, Jiang et al., put forward a novel multi-scale analysis method called hierarchical analysis, which can reveal the inherent fault characteristics of a vibration signal in both low- and high-frequency components [29,30]. Derived from hierarchical analysis, hierarchical sample entropy (HSE) [31], hierarchical fuzzy entropy (HFE) [32], and hierarchical permutation entropy (HPE) [33] were proposed to extract fault characteristics.

Inspired by the advantages of hierarchical analysis and the *AAPE* algorithm, the *HAAPE* method for rolling bearings is proposed, and it includes the following two aspects: performance degradation assessment and fault diagnosis. In the performance degradation assessment stage, *HAAPE* is employed as an index to quantify the running state of the bearing in its run-to-failure life cycle, and Chebyshev’s inequality theory is applied to establish a health threshold to judge the bearing performance trend between the healthy state and abnormal state. In the fault diagnosis stage, *HAAPE* is used after the LCM-SVD procedure to identify the bearing failure characteristics. Two experimental cases demonstrate that the proposed *HAAPE* can effectively extract the fault characteristics, and the experimental results show that *HAAPE* has superiority over existing methods in the prediction of performance degradation trend and fault diagnosis of rolling bearings.

The remainder of this article is constructed as follows: Section 2 introduces the related theoretical basis and the fundamentals of *HAAPE*. Simulation signals are used to verify the effectiveness of the proposed method in Section 3. The experimental results are arranged in Section 4, which includes two experimental cases, namely, case 1 and case 2. Finally, several conclusions are drawn in Section 5.

## 2. Methodologies

### 2.1. Amplitude-Aware Permutation Entropy (*AAPE*)

A one-dimensional time series *X*(*i*), *i* = 1, 2, …, *N* is denoted as the original signal. At any time point *j*, the *m* dimensional reconstruction vector $X_{j}^{m,\tau }$ with the delay time *τ* can be obtained as follows:

(1) $$X_{j}^{m,\tau }=\left[\begin{array}{cccc} x_{\left(1\right)} & x_{\left(1+\tau \right)} & \cdots & x_{\left[1+(m-1)\tau \right]}\\ x_{\left(2\right)} & x_{\left(2+\tau \right)} & \cdots & x_{\left[2+(m-1)\tau \right]}\\ x_{\left(j\right)} & x_{\left(j+\tau \right)} & \cdots & x_{\left[j+(m-1)\tau \right]}\\ & & \vdots & \\ x_{\left(k\right)} & x_{\left(k+\tau \right)} & \cdots & x_{\left[k+(m-1)\tau \right]} \end{array}\right],\;j=1,\;2,\;\ldots ,\;k=N-(m-1)\tau$$

The elements in the *j*-th reconstructed vector are arranged in ascending order as shown in Equation (2).

(2) $$x[i+(j_{1}-1)\tau ]\leq x[i+(j_{2}-1)\tau ]\leq \cdots \leq x[i+(j_{m}-1)\tau ]$$

where *j*_1_, *j*_2_, …, *j_m_* are the index of the column of each element in the reconstructed vector. Hence, there are *m*! potential ordinal patterns, of which the *d*-th permutation is marked as *π_d_*. The occurrence times of each *π_d_* are calculated to obtain their occurrence probability. Finally, based on the definition of Shannon entropy, PE can be defined.

(3) $$PE(x,m,\tau )=-\sum_{\pi_{d}=1}^{m!}p(\pi_{d})\mathrm{In}p(\pi_{d})$$

**Figure 1:**
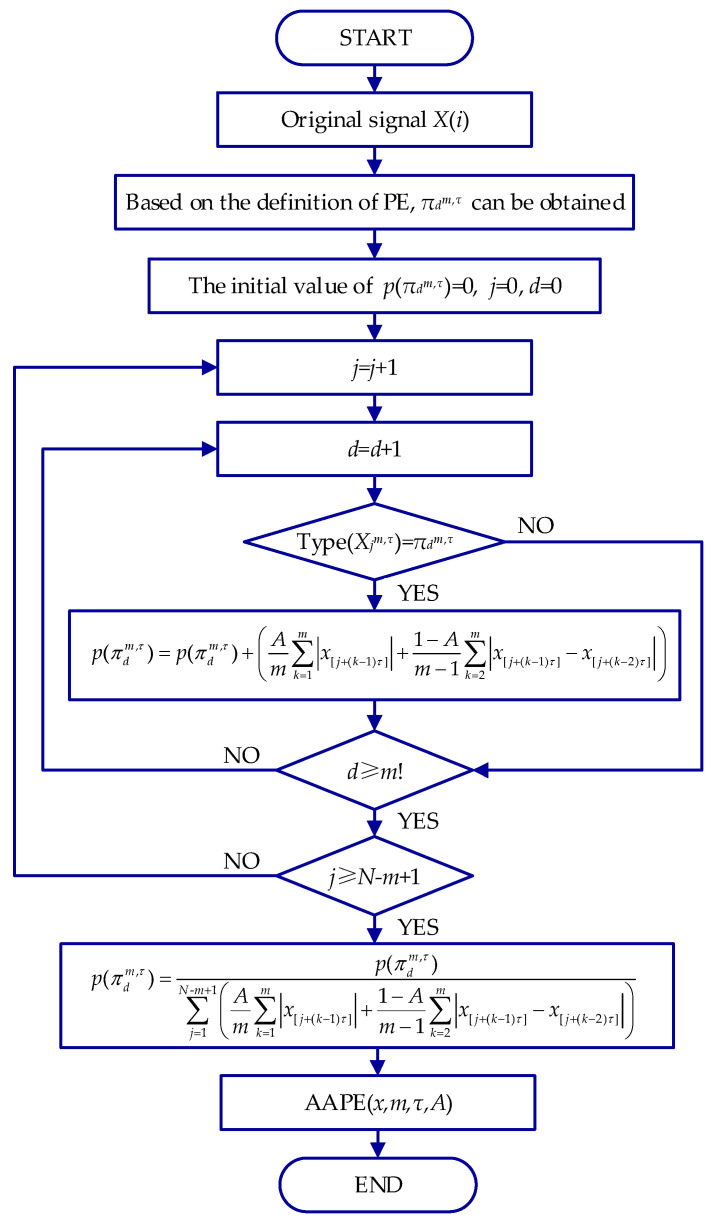
Flowchart of the *AAPE*.

However, there are two main shortcomings in the application process: (1) PE only considers the sequence number structure of the time series rather than the amplitude difference between sequential samples, which results in the loss of some valuable characteristics. (2) Under the appearance of equal amplitude, the difference in permutation patterns cannot be distinguished using PE. The *AAPE* based on PE addresses the two problems mentioned above. A flowchart of *AAPE* is given in Figure 1.

Supposing that the initial value of *p*($\pi_{d}^{m,\tau }$) is 0, for the original signal *X*, as *j* increases from 1 to *N* − *m* + 1, *p*($\pi_{d}^{m,\tau }$) should be renewed whenever $\pi_{d}^{m,\tau }$ appears.

(4) $$p(\pi_{d}^{m,\tau })=p(\pi_{d}^{m,\tau })+\left(\frac{A}{m}\sum_{k=1}^{m}\left|x_{\left[j+(k-1)\tau \right]}\right|+\frac{1-A}{m-1}\sum_{k=2}^{m}\left|x_{\left[j+(k-1)\tau \right]}-x_{\left[j+(k-2)\tau \right]}\right|\right)$$

where *A* (*A* ∈ [0,1]) stands for the correlation adjustment coefficient, which is related to the mean value and deviation between consecutive amplitudes.

Then, the probability of *p*($\pi_{d}^{m,\tau }$) of the time series is given by Equation (5).

(5) $$p(\pi_{d}^{m,\tau })=\frac{p(\pi_{d}^{m,\tau })}{\sum_{j=1}^{N-m+1}\left(\frac{A}{m}\sum_{k=1}^{m}\left|x_{\left[j+(k-1)\tau \right]}\right|+\frac{1-A}{m-1}\sum_{k=2}^{m}\left|x_{\left[j+(k-1)\tau \right]}-x_{\left[j+(k-2)\tau \right]}\right|\right)}$$

The *AAPE* calculation of the original signal *X* after normalization can be calculated by Equation (6).

(6) $$AAPE(x,m,\tau ,A)=\frac{-\sum_{\pi_{{}^{d}}^{m,\tau }=1}^{m!}p(\pi_{{}^{d}}^{m,\tau })\mathrm{In}p(\pi_{{}^{d}}^{m,\tau })}{\mathrm{In}(m!)}$$

### 2.2. The Least Common Multiple in Singular Value Decomposition (LCM-SVD)

The SVD, a matrix orthogonalization decomposition method, has been widely used in fault diagnosis analysis. A one-dimensional vibration time series needs to be reconstructed into a matrix, and the Hankel matrix is commonly applied in various formats. Given the original signal *X*(*i*), *i* = 1, 2, …, *N*, the Hankel matrix *H*_m×n_ can be reconstructed as

(7) $$H_{m\times n}=\left[\begin{array}{cccc} X(1) & X(2) & \cdots & X(n)\\ X(2) & X(3) & \cdots & X(n+1)\\ & & \vdots & \\ X(m) & X(m+1) & \cdots & X(N) \end{array}\right]$$

where *1* < *n* < *N*, *m* = *N* − *n* + 1.

The SVD of *H*_*m*×*n*_ can be defined as follows:

(8) $$H_{m\times n}=U\sum V^{T}=[u_{1},u_{2},\cdots ,u_{q}]\left[\begin{array}{cccc} \sigma_{1} & 0 & \cdots & 0\\ 0 & \sigma_{2} & \ddots & \vdots \\ \vdots & \ddots & \ddots & 0\\ 0 & \cdots & 0 & \sigma_{q} \end{array}\right]\left[\begin{array}{c} v_{1}\\ v_{2}\\ \vdots \\ v_{q} \end{array}\right]$$

where the left singular vectors *U_m_*_×*m*_ and right singular vectors *V_n_*_×*n*_ are orthogonal matrices, and *Σ_m_*_×*n*_ is a diagonal matrix composed of singular values (*σ*_1_ ≥ *σ*_2_ ≥ …≥ *σ_q_* ≥ 0, *q* = min(*m*, *n*)).

**Figure 2:**
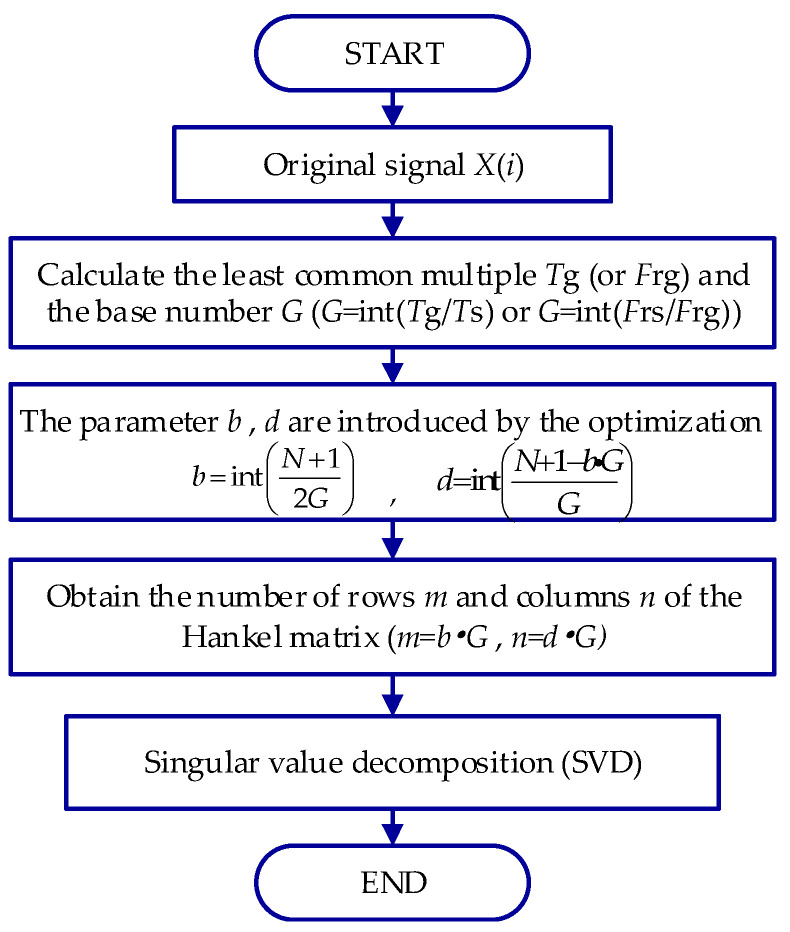
Flowchart of LCM-SVD.

For a noisy signal, the matrix dimension affects the denoising accuracy of SVD in fault identification. Under the matrix dimension determined by the least common multiple in SVD (LCM-SVD) method, the waveform error of the denoising results is much smaller than that of the traditional maximum dimension method [16]. Thus, we can obtain a well denoised signal via the LCM-SVD method. A flowchart of the LCM-SVD analysis process is shown in Figure 2, and the three steps are presented below.

Firstly, the least common multiple *T_g_* is calculated by the periods of all frequency components. In the envelope spectrum analysis of the rotor system, each frequency of higher harmonics has a prominent frequency and is an integer multiple of the rotational frequency. Therefore, the least common multiple *F_rg_* of each frequency component in the signal is the period of rotational frequency.

Then, a base number *G* is the ratio of the sampling period *T_s_* (or sampling frequency *F_rs_*) and the least common multiple *T_g_* (or *F_rg_*). To maximize the dimension of the Hankel matrix, the parameters *b* and *d* are introduced by the optimization computation.

Finally, the suitable row *m* and column *n* of the Hankel matrix form are determined to prepare for SVD.

### 2.3. Hierarchical Amplitude-Aware Permutation Entropy (*HAAPE*)

**Figure 3:**
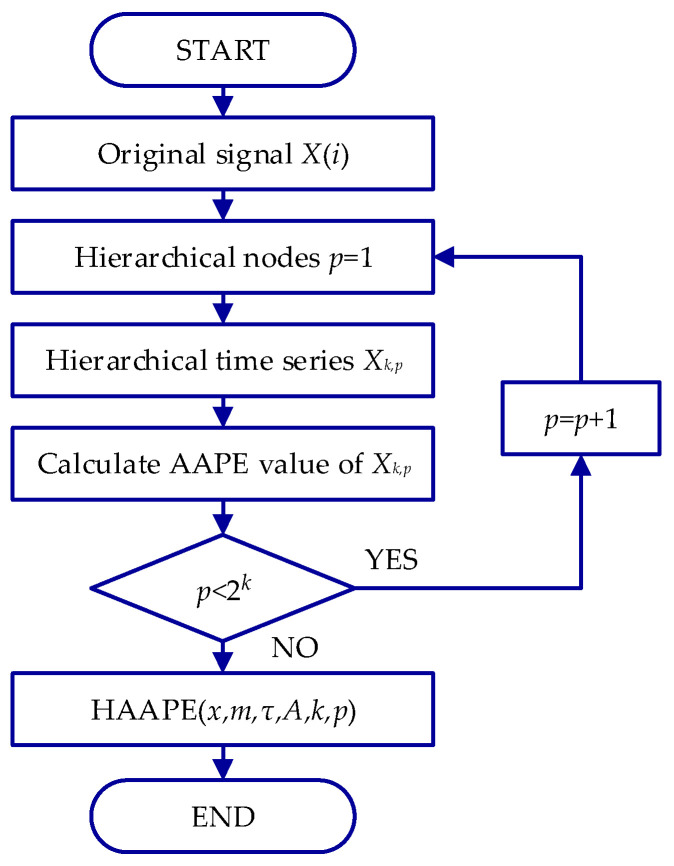
Flowchart of the *HAAPE* method.

*HAAPE* is derived from the hierarchical analysis method and the *AAPE* algorithm, and its flowchart is shown in Figure 3. The detailed calculation procedure of *HAAPE* can be described as follows:

(1) For a one-dimensional time series *X*(*i*), *i* = 1, 2,…, *N*, two operators *Q*_0_ and *Q*_1_ are defined as

(9) $$\{\begin{array}{c} Q_{0}(x)=\frac{x(2j)+x(2j+1)}{2}\\ Q_{1}(x)=\frac{x(2j)-x(2j+1)}{2} \end{array},\;j=1,\;2,\;\ldots ,\;2^{n-1}$$

where *N* = 2*^n^*, and *n* is a positive integer, which represents the *n*-th level of decomposition. The averaging operator *Q*_0_ and difference operator *Q*_1_ describe the low- and high-frequency components of the signal, respectively. Accordingly, the original time series can be rebuilt by *Q*_0_ and *Q*_1_.

(10) $$X=\left\{\left(Q_{0}{\left(x\right)}_{j}+Q_{1}{\left(x\right)}_{j}\right),\;\left(Q_{0}{\left(x\right)}_{j}-Q_{1}{\left(x\right)}_{j}\right)\right\},\;j=1,\;2,\;\ldots ,\;2^{n-1}$$

(2) The matrix *Q_j_* operator is defined when *j* = 0 or *j* = 1 as follows:

(11) $$Q_{j}(x)={\left[\begin{array}{ccccccc} \frac{1}{2} & {\frac{\left(-1\right)}{2}}^{j} & 0 & 0 & \cdots & 0 & 0\\ 0 & 0 & \frac{1}{2} & {\frac{\left(-1\right)}{2}}^{j} & \cdots & 0 & 0\\ & & & & \ddots & & \\ 0 & 0 & 0 & 0 & \cdots & \frac{1}{2} & {\frac{\left(-1\right)}{2}}^{j} \end{array}\right]}_{2^{n-1}\times 2^{n}}$$

(3) To conduct a hierarchical analysis of a time series, the operators *Q*_0_ and *Q*_1_ are applied repeatedly in step 2. To describe each node of the *k*-th layer in the hierarchical analysis, we introduce the positive integer parameter *p* and a vector [*ζ*_1_, *ζ*_2_, …, *ζ_k_*]. The relationship between *p* and the vector is calculated as follows:

(12) $$p=\sum_{i=1}^{k}2^{k-i}\zeta_{i}$$

That is, when *k* ∈ $Z_{N}^{+}$ is fixed, *p* corresponds to the sole vector [*ζ*_1_, *ζ*_2_, …, *ζ_k_*] ∈ {0, 1} by Equation (12). For example, when *k* = 3 and *p* = 7, the unique vector [*ζ*_1_, *ζ*_2_, *ζ*_3_] = [1, 1, 1].

(4) The sub-signal with node *p* component of the *k*-th layer decomposition in time series *X_k,p_* can be obtained by Equation (13). Therefore, the high-frequency components *X*_3,7_ can be denoted as $Q_{1}^{3}$·$Q_{1}^{2}$·$Q_{1}^{1}$·*X*(*i*).

(13) $$X_{k,p}=Q_{{}_{\zeta_{k}}}^{k}\cdot Q_{{}_{\zeta_{k-1}}}^{k-1}\cdot \ldots \cdot Q_{{}_{\zeta_{1}}}^{1}\cdot X(i)$$

**Figure 4:**
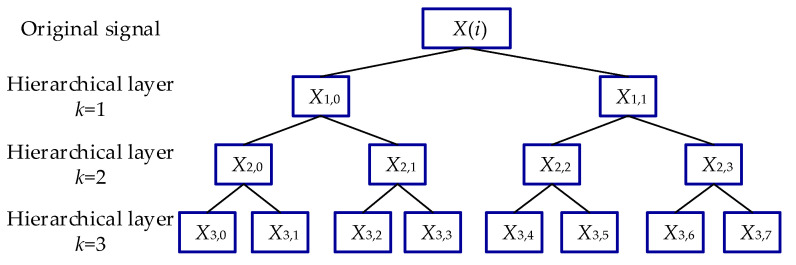
Hierarchical decomposition of original signal with three layers.

(5) Finally, under the hierarchical layer *k*, the *HAAPE* value of the original signal X(i) can be expressed by the *AAPE* value of each layer node component.

(14) $$HAAPE(x,m,\tau ,A,k,p)=AAPE(x_{k,p},m,\tau ,A)$$

where *m* is the embedding dimension, and *τ* is the time delay. The schematic diagram of the hierarchical analysis contains three layers, as shown in Figure 4.

Five parameters need to be determined before calculating the *HAAPE* algorithm: signal length *N*, embedding dimension *m*, time delay *τ*, correlation adjustment coefficient *A*, and hierarchical decomposition layer *k*. Specifically, the embedding dimension *m* is associated with the signal length *N* (*N* = 2048). If the value of *m* is set too small, the phase space reconstructed vector will lose its usefulness and effectiveness. Conversely, if *m* is set too large, the phase space reconstructed vector will fail to reveal the change between time series distinctly. Generally, the embedding dimension *m* is 3–7 [34]. Time delay *τ* is normally set to 1. Correlation adjustment coefficient *A* is usually set to 0.5 since the significance of the average amplitude is equal to the difference of the amplitude values. However, *A* << 0.5 is recommended when the difference between two consecutive time series is more important than the average amplitude [22]. For the selection of the hierarchical decomposition layer *k*, the length of the time series is shortened with an increase in the *k* value. However, if the hierarchical decomposition layer *k* is smaller, some critical low- and high-frequency characteristics cannot be obtained, so the layer number *k* = 3 is usually selected [35].

## 3. Numerical Simulation Analysis

**Figure 5:**
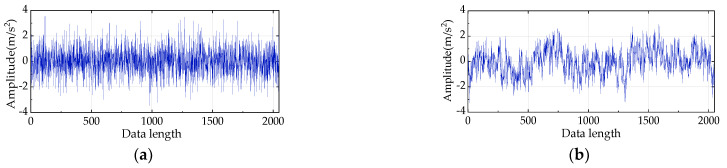
(**a**) Temporal waveform of WGN, and (**b**) temporal waveform of 1/f noise.

In this section, *HAAPE*, IMAAPE, MAAPE, and HPE are selected for performance comparison. The 50 sets’ independent random WGN and 1/f noise containing 2048 data points are adopted to observe four entropy trends. Figure 5a,b show the randomly selected temporal waveforms of WGN and 1/f noise, respectively. It can also be seen in Figure 5a that WGN is a random distribution and has a stable complexity in the period. In contrast, 1/f noise in Figure 5b has a more complex structure and contains more mode information. Consequently, 1/f noise is more complex than WGN regarding structure, whereas WGN is greater than 1/f noise in terms of the quality of irregularities. Theoretically, the entropy value of WGN will be stable and higher than that of 1/f noise.

**Figure 6:**
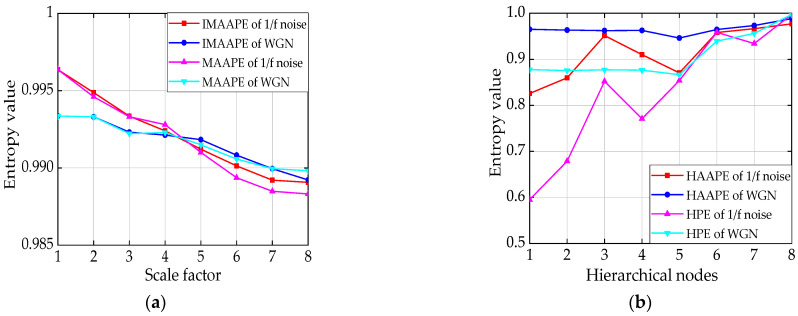
(**a**) Average of 50 sets of values of IMAAPE and MAAPE, and (**b**) average of 50 sets of values of *HAAPE* and HPE.

**Figure 7:**
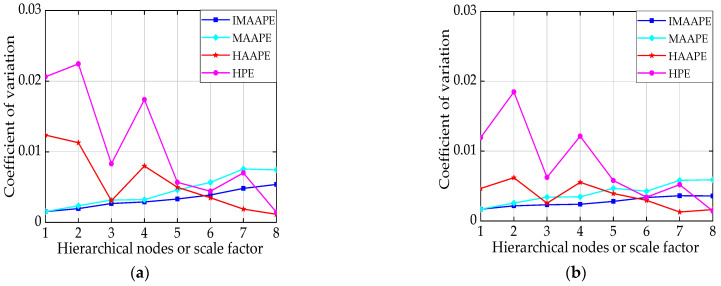
(**a**) CV values of four entropies for 1/f noise, and (**b**) CV values of four entropies for WGN.

To ensure comparability and effectiveness, the parameters of the four various entropy algorithms are defined as the following: embedding dimension *m* = 3, time delay *τ* = 1, adjustment coefficient *A* = 0.5, scale factor *t* = 8, and layer number *k* = 3 (contains eight nodes). Then, the four entropies of WGN and 1/f noise are normalized, and the averages of 50 sets of values are displayed in Figure 6. In Figure 7, to eliminate the influence of the measurement scale, the coefficient of variation (CV) is used to judge the distribution degree of the four entropies, where the CV value is equal to the standard deviation divided by the mean value. The simulation results are defined as follows:

(1) In Figure 6, the *HAAPE* and HPE values of WGN are greater than those of 1/f noise, which is in accordance with the theoretical analysis. The IMAAPE and MAAPE values of WGN are greater than those of 1/f noise in the last four scales (scale = 5,6,7,8).

(2) The *HAAPE* and HPE value curves can separate WGN noise and 1/f noise signals well, while both the IMAAPE and MAAPE value curves overlap on most scales and have poor separability in Figure 6.

(3) As can be seen in Figure 7, whether WGN or 1/f noise, the CV value of IMAAPE is lower than that of MAAPE on all scales. This indicates that IMAAPE is more stable than MAAPE. Meanwhile, the CV value of *HAAPE* is smaller than that of HPE in whole scales, which illustrates that the *AAPE* algorithm is superior to feature extraction.

(4) In Figure 7, as the scale factor (or hierarchical nodes) increases, the CV value of IMAAPE steadily increases, while *HAAPE* gradually decreases, indicating that the stability is significantly improved. Hierarchical analysis has an advantage over the traditional multi-scale analysis because the hierarchical analysis can simultaneously utilize the signal’s low- and high-frequency components.

Accordingly, compared with the other three methods (IMAAPE, MAAPE, and HPE), *HAAPE* can extract entire information and detect the dynamic trend of complex time series.

## 4. Experimental Data Analysis

### 4.1. Experimental Setup

The data sets in this section were published by the Center on Intelligent Maintenance Systems (IMS), the University of Cincinnati [36]. The data sets are used to conduct two cases, one for trend analysis of rolling bearing performance degradation to explicate the effectiveness of the *HAAPE* algorithm and the other one for a feature selection strategy based on *HAAPE* after LCM-SVD identifies valid fault characteristics.

**Figure 8:**
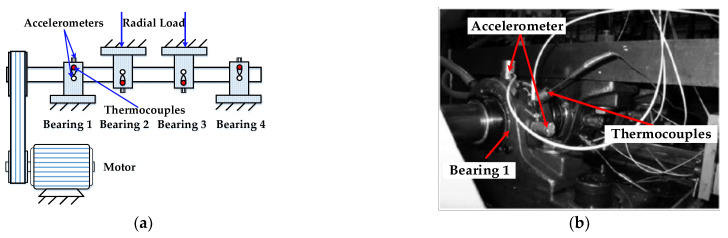
(**a**) The experimental bench’s schematic diagram and (**b**) its physical diagram.

The experimental bench’s schematic diagram and physical diagram are shown in Figure 8; four ZA-2115 type double-row roller bearings are installed on the single shaft. The radial load of the middle bearings, namely, bearings 2 and 3, is 6000 lbs, and bearings 1 and 4 play the supporting role. The shaft is driven by a motor, and its rotating speed is stable at 2000 r/min. During operation, the vibration data are collected by the PCB 353B33 high-precision ICP vibration sensor at a sampling frequency of 20 kHz (each sampling time is 1 s) every 10 min, collecting 20,480 data points. The experiment obtains three experimental data sets, ranging from the installation of the new bearing to complete failure. We use the second set for experimental analysis. The second set has 984 data sets, and each data set contains the vibration signals of the bearings in one direction. The experiment runs for 163.83 h, and the results show that the outer ring of bearing 1 fails as a result of disassembling and inspecting bearing 1 [37]. Table 1 presents the structural characteristics of ZA-2115 and its ball pass frequency outer (BPFO).

### 4.2. Case 1: Rolling Bearing Performance Degradation Trend Analysis

The trend analysis of rolling bearing performance degradation investigates the correlation between the healthy operational state and the whole life process of test signals. The RMS, kurtosis, *HAAPE*, IMAAPE, HPE, and PE methods are selected for comparison. The common analysis indexes used to evaluate the whole life cycle are the RMS and kurtosis values. The experimental data are divided into ten non-overlapping groups, and the average entropy value of the ten groups is used as the output result.

**Figure 9:**
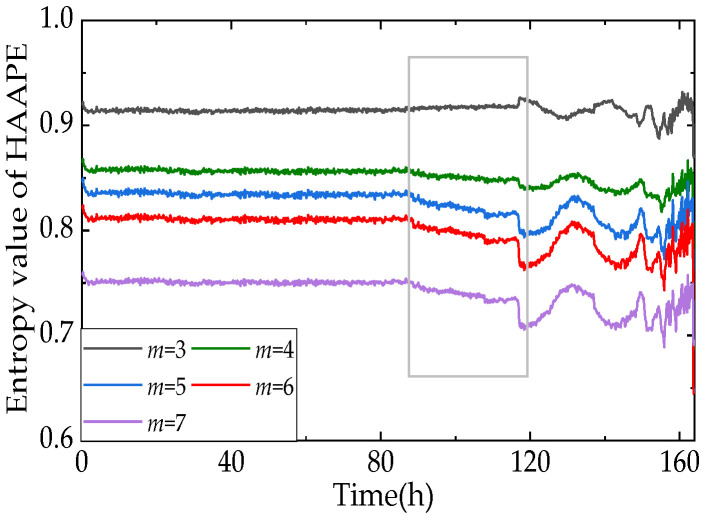
The influences of embedding dimensions on *HAAPE*.

The amplitude value between two consecutive sampling points is more valuable than the average amplitude when employing performance degradation trend analysis, so the correlation adjustment coefficient *A* is set to 0.02 in this section. The scale factor should be as small as possible to avoid losing features in the multi-scale analysis [38]. Thus, the hierarchical decomposition layer *k* is fixed to 2, and scale factor *t* = 4. Figure 9 depicts the effects of the embedding dimension on *HAAPE*. With an increase in the embedding dimension *m*, the performance degradation trend notably grows in the gray rectangle. Nevertheless, there is no significant difference in effect between *m* = 6 and *m* = 7. Meanwhile, in the computation process, we find that *m* = 6 takes less time to calculate than *m* = 7, so m = 6 is best.

The whole life of a rolling bearing can be divided into two conditions: a healthy state and an abnormal one. In practical engineering applications, we monitor the two conditions to obtain the degradation trend of the rolling bearing. Chebyshev’s inequality theory is introduced to determine the effective health threshold, which is defined as follows:

(15) $$P\left\{\left|X-\mu_{h}\right|\geq \varepsilon_{h}\right\}\leq \frac{\sigma_{h}^{2}}{\varepsilon_{h}^{2}}\;\mathrm{or}\;P\left\{\left|X-\mu_{h}\right|<\varepsilon_{h}\right\}\geq 1-\frac{\sigma_{h}^{2}}{\varepsilon_{h}^{2}}$$

where *X* stands for the series of *HAAPE* values under healthy conditions, and *μ_h_* and *σ_h_* represent the mean value and standard deviation of *X*, respectively. According to Equation (15), when *ε_h_* is set to 5*σ_h_*, the entropy value of a bearing is about *μ_h_* ± 5*σ_h_* in the healthy condition. In general, the test bearing remains healthy during the first one-quarter of the time period [39,40]. Therefore, we apply the first 50 h of indicator data to calculate the health threshold through Equation (15) to minimize errors. The *HAAPE* value of the rolling bearing data series exceeds the health threshold, which confirms that the rolling bearing is under abnormal conditions with 96% confidence probability.

**Figure 10:**
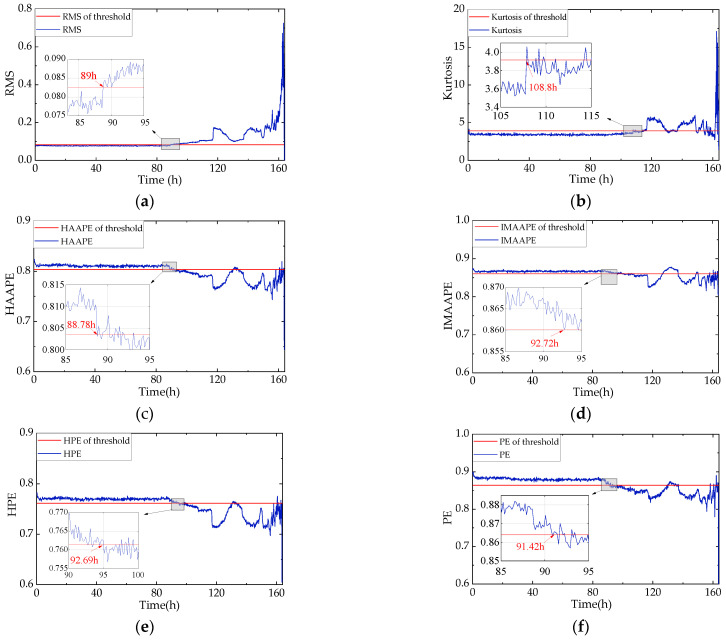
Six indicators with performance degradation trend analysis over whole lifetime. (**a**) RMS; (**b**) Kurtosis; (**c**) *HAAPE*; (**d**) IMAAPE; (**e**)HPE and (**f**) PE.

RMS and the kurtosis value are sensitive to bearing degradation [39], so RMS and kurtosis are shown as evaluation indexes in Figure 10a,b, respectively. It can be seen in Figure 10a that when early failure occurs, the RMS values surpass the health threshold at nearly 89 h, and the change range is relatively small. Meanwhile, the kurtosis values indicate that the health threshold and curve intersect occur approximately 20 h later than those of the RMS index at 108.8 h in Figure 10b. This also reveals that kurtosis is insensitive to early bearing performance degradation.

To investigate the trend analysis of the *HAAPE* algorithm, the algorithms IMAAPE, HPE, and PE are utilized for the comparison experiment. As shown in Figure 10c, the *HAAPE* values exceed the health threshold at 88.78 h and display a sharp drop, indicating a sudden change in the bearing running state. This demonstrates that the *HAAPE* algorithm successfully extracts the fault features of the rolling bearings and detects the early performance decline trend, which occurs earlier than the RMS index at about 13 min. In addition, the value of *HAAPE* maintains a stable state in the healthy stage; the entropy retains small values; and the curve varies when a bearing fault occurs, which means that the vibration signals of the healthy state are more complex and random than that of the abnormal state. Therefore, the *HAAPE* algorithm is sensitive to early bearing fault detection, and it draws the degradation trend of bearings effectively and accurately. In Figure 10d,e, both IMAAPE and HPE values indicate the occurrence of degradation at 93 h, about 3 h later than *HAAPE*. In addition, the range of entropy change is relatively smaller when detecting early failure, which shows that the above two algorithms have poor sensitivity to early failure. Figure 10f shows that the intersection of the health threshold and the PE curve is at 91 h, which is earlier than IMAAPE and HPE but later than *HAAPE*.

In summary, *HAAPE* can extract fault characteristics of rolling bearings and assess performance degradation process, which has a profound significance for the life prediction of rolling bearings.

### 4.3. Case 2: The Feature Selection Strategy Based on *HAAPE* after LCM-SVD

**Figure 11:**
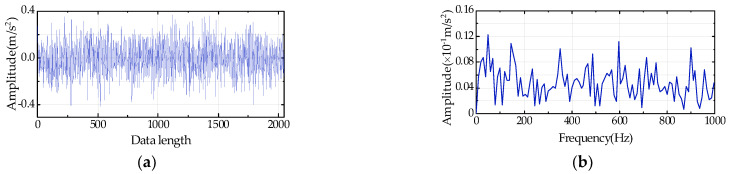
(**a**) Time-domain waveform, and (**b**) Hilbert envelope spectrum of the 533rd data group with Gaussian noise.

In practical conditions, the operating environment of the bearing is often buried in noise. To verify the effectiveness of the proposed method, the 533rd, 534th, and 700th data groups are taken as analysis objects in this case and random Gaussian noise with SNR = 1 dB is added to the original vibrational signals. The 533rd and 534th data groups are the bearing vibration data when an early fault occurs, while the 700th data group is collected when bearing failure aggravates. Figure 11 shows the time-domain waveform and envelope spectrum of the 533rd data group. Figure 11a exhibits a random group signal time-domain waveform with a data length of 2048. Figure 11b displays the confusing and complex Hilbert envelope spectrum, and the fault frequency cannot be extracted because of noise in the time series. Consequently, this case requires envelope spectrum analysis to be carried out after LCM-SVD denoising.

The difference spectrum unilateral maxima of singular value (DSUMSV) determines the effective rank order number by, from right to left, selecting the coordinate value corresponding to the highest difference of the peak compared with its neighbor one. This case adopts the DSUMSV to describe *HAAPE*’s accuracy of identification. To validate the superiority of the *HAAPE* algorithm, the IMAAPE algorithm is compared in this case to a related analysis, and the selection of parameters (*N*, *m*, *τ*, *A*, *k*) refers to those presented in Section 2.3.

**Figure 12:**
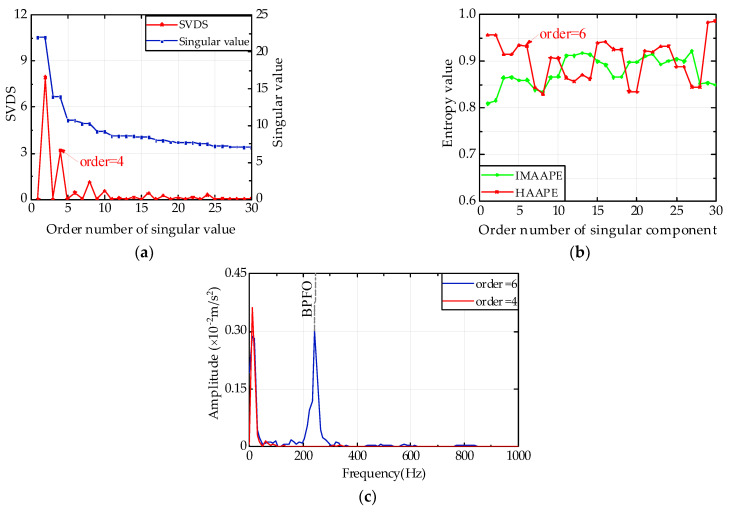
Analysis diagram of 533rd data group with Gaussian noise. (**a**) DSUMSV, (**b**) *HAAPE* and IMAAPE, and (**c**) Hilbert envelope spectrum.

As shown in Figure 12a, there are three peaks in total in the singular value difference spectrum (SVDS), and the second maximum peak is in the fourth coordinate using the DSUMSV principle. Furthermore, its value is the highest difference from the maximum peak, which means that the maximum sudden change in the singular value happens in the fourth coordinate. This change shows the boundary of singular values between the signal and noise, so the effective rank order is four. In Figure 12b, the *HAAPE* values of the first 30 singular components are calculated, and every two values in the first six entropies nearly equal each other. Therefore, the effective rank order is set to six. The values of IMAAPE have no rules at all in Figure 12b. In Figure 12c, the rebuilt signal is analyzed by envelope analysis, where the Fourier transform of the envelope signal is multiplied by the Hann window function. Less useful frequency information can be extracted when the rank order is equal to four, while a frequency position stands out on the curve at 244.1 Hz when the order equals six, which is approximately equal to the fault frequency of the outer ring.

**Figure 13:**
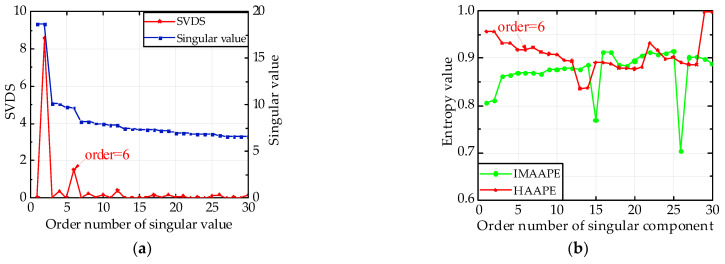

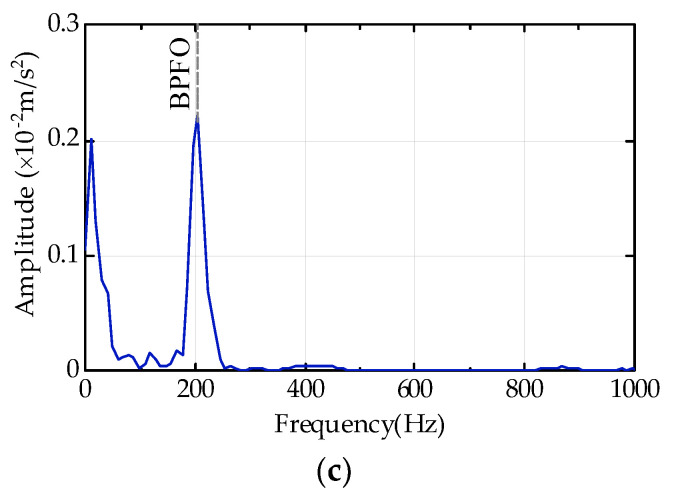
Analysis diagram of 534th data group with Gaussian noise. (**a**) DSUMSV, (**b**) *HAAPE* and IMAAPE, and (**c**) Hilbert envelope spectrum.

In Figure 13a,b, the DSUMSV method and *HAAPE* algorithm results show that the first six coordinates are identified as the effective rank order number. Meanwhile, with difficulty, we can observe the regularity of the IMAAPE values change in Figure 13b. As shown in Figure 13c, when the rank order is equal to six, the envelope analysis curve has a prominent peak at 205.1 Hz, which further suggests the possible potential damage to the outer ring. Therefore, the comparative analysis reveals that *HAAPE* is better than IMAAPE in characteristic frequency recognition.

**Figure 14:**
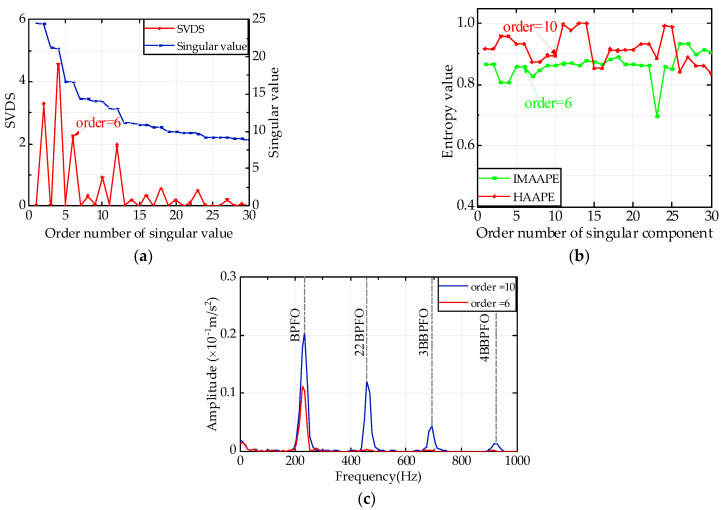
Analysis diagram of 700th data group with Gaussian noise. (**a**) DSUMSV, (**b**) *HAAPE* and IMAAPE, and (**c**) Hilbert envelope spectrum.

Figure 10 shows the aggravation of the bearing failure, especially in the 700th data group. Figure 14a indicates that the second maximum peak is the highest difference from the maximum peak, while the second maximum peak is located in the sixth coordinate. Hence, the effective rank order is set to six. According to Figure 14b, the *HAAPE* values of the first ten coordinates show a specific tendency, while the IMAAPE algorithm presents regularity in the first six coordinates. Consequently, the effective rank order is set to 10. In Figure 14c, when the effective rank order is six, only the fault frequency of the outer ring is found, and there is no more multiplier information about the fault frequency. Under the effective rank order of 10, the envelope curve has 3 prominent peaks at the fault frequency of 234.4 Hz, dual-frequency of 459 Hz, and tri-frequency of 693.4 Hz. Based on the above analysis, the failure of the bearing outer ring can be confirmed.

## 5. Conclusions

To evaluate the performance degradation trend of rolling bearings and to improve the accuracy of extracting useful fault features, a nonlinear dynamic signal analysis method called *HAAPE* based on *AAPE* is proposed to weigh the complexity of the time series composed of the fault vibration signal. Numerical simulation signals and experimental data verify the effectiveness of *HAAPE*. The following several conclusions can be drawn:

(1) Compared with the existing algorithms IMAAPE, MAAPE, and HPE, *HAAPE* has better stability and accuracy. It solves the issues arising from multi-scale entropy, which only considers the low-frequency components of the signal and ignores some information in the high-frequency components. Accordingly, *HAAPE* can extract more useful fault characteristics at both low- and high-frequencies from the vibration signal of rolling bearings.

(2) The results of experimental case 1 indicate that the proposed *HAAPE* can efficiently reflect the bearing performance degradation trend. Additionally, this approach is more sensitive to the early identification of failures compared to the other five methods. Hence, the *HAAPE* algorithm is an effective index for assessment performance degradation.

(3) Case 2 verifies that the *HAAPE* algorithm accurately decides the effective rank order. Especially after LCM-SVD denoising, *HAAPE* can perfectly identify the fault characteristics of the bearing using a few feature vectors, which reduces information redundancy and calculative burden.

## Figures and Tables

**Table 1:** The structural characteristic of ZA-2115 and its ball pass frequency outer ring.

Contact Angle	Pitch Diameter	Roller Diameter	Roller Number	BPFO
15.17°	71.5 mm	8.4 mm	16	236.4 Hz

## Data Availability

The experimental data is available from the IMS Bearing Data deposited in https://ti.arc.nasa.gov/tech/dash/groups/pcoe/prognostic-data-repository/ (accessed on 2 January 2022).
